# Estrogen accelerates the resolution of inflammation in macrophagic cells

**DOI:** 10.1038/srep15224

**Published:** 2015-10-19

**Authors:** Alessandro Villa, Nicoletta Rizzi, Elisabetta Vegeto, Paolo Ciana, Adriana Maggi

**Affiliations:** 1 Center of Excellence on Neurodegenerative Diseases and Department of Pharmacological and Biomolecular Sciences, University of Milan, Milan, Italy

## Abstract

Although 17β-estradiol (E_2_) anti-inflammatory activity has been well described, very little is known about the effects of this hormone on the resolution phase of the inflammatory process. Here, we identified a previously unreported ERα-mediated effect of E_2_ on the inflammatory machinery. The study showed that the activation of the intracellular estrogen receptor shortens the LPS-induced pro-inflammatory phase and, by influencing the intrinsic and extrinsic programs, triggers the resolution of inflammation in RAW 264.7 cells. Through the regulation of the SOCS3 and STAT3 signaling pathways, E_2_ facilitates the progression of the inflammatory process toward the IL10-dependent “acquired deactivation” phenotype, which is responsible for tissue remodeling and the restoration of homeostatic conditions. The present study may provide an explanation for increased susceptibility to chronic inflammatory diseases in women after menopause, and it suggests novel anti-inflammatory treatments for such disorders.

The hypothesis that estrogens may play a role in protection against chronic inflammatory diseases stems from a large body of clinical and preclinical evidence that has demonstrated that *a)* the etiology and course of chronic inflammatory diseases is influenced by the menstrual cycle and pregnancy^1^,^2^,^3^; *b)* a significant increase in the incidence of disorders characterized by a strong inflammatory component (e.g., osteoporosis, atherosclerosis, diabetes and other metabolic diseases, arthritis and others) is a hallmark of the cessation of ovarian functions^4^; and *c)* fertile female mammals are less prone than males to these diseases^5^,^6^. Indeed, circulating estrogens were shown to target cells of monocyte lineage and to mitigate several of the aspects of the native immune response. Our prior results in rodent cells showed that macrophages express the classical intracellular receptors α and β (ERα and ERβ)^7^. In addition, other authors demonstrated that also the membrane-bound G-protein-coupled estrogen receptor (GPR30/GPER-1) is expressed in murine macrophages^8^. The ERα-bound estrogen was shown to decrease the synthesis of pro-inflammatory compounds^9^,^10^, to control mitochondrial mitosis^11^ and respiratory function^12^, to induce the degradation of damaged proteins through the proteasome^13^, and to diminish monocyte apoptosis and activation of hypoxic genes^14^. At a molecular level, the hormone-activated ERα was shown to impair NF-κB transcriptional activity by preventing its nuclear translocation in the presence of strong inflammatory stimuli (such as lipopolysaccharide, LPS). In this case, the estrogen-dependent prevention of NF-κB activation did not require *de novo* protein synthesis to modify IκBα degradation, and it occurred through a direct interaction between the hormone-activated ERα and phosphatidylinositol 3-kinase^10^. In fact, the intracellular ERα and ERβ, in addition to acting as hormonally regulated transcription factors, may also interact with cytoplasmic and nuclear proteins responsible for signal transduction and interfere with their functions^15^. Recent studies have suggested that, in addition to intracellular receptors, the membrane-bound GPR30 receptor may be involved in estrogen anti-inflammatory action by pinpointing GPR30-specific agonists that regulate the expression of the cell-surface of Toll-like receptor 4 (TLR4) in macrophages^8^. Monocytes/macrophages are exceptionally plastic cells able to adopt the context-specific phenotypes necessary to protect the host from harmful events and to reinstate tissue homeostasis when the inflammatory reaction is over. This is achieved with significant changes in the transcriptome and proteome: inflammatory stimuli (derived from microbes, damaged tissues or activated lymphocytes) induce macrophages to acquire the pro-inflammatory polarization or M1 consisting of the rapid activation of NF-κB and the synthesis and secretion of pro-inflammatory cytokines (e.g., TNFα, IL1β, IL6, IL12, and IL23) and chemokines (particularly monocyte chemotactic protein-1, MCP1). These inflammatory compounds are responsible for the increased expression of cytotoxic mediators such as inducible nitric oxide synthase (iNOS). They are also responsible for increased expression of enzymes relevant for the phagocytosis of damaged tissues or microorganisms and for the migration and further recruitment of macrophages. This *sequelae* of events aimed at the rapid and efficient removal of the inflammatory stimuli is often the cause of severe damage at the site of inflammation; thus, a well-balanced immune response ends with the repair and functional recovery of all damaged tissues. This “resolution phase” is carried out by macrophages in concert with the host tissue and other immune cells as lymphocytes and occurs by acquiring the conventionally named alternative activation (AA) state or M2a polarization^16^ in which macrophages further prompt the synthesis and secretion of specific anti-inflammatory cytokines (mainly IL4 and IL13) by T lymphocytes to finally repress the production of pro-inflammatory cytokines (like TNFα, IL12, and IL1β) and iNOS. Recent studies have shown that macrophages may also activate an intrinsic program that converts them to the anti-inflammatory phenotype^17^. The dampening of the inflammatory response is associated with the “acquired deactivation” (AD) or M2c phenotype, characterized by the production of IL10 and TGFβ^16^, compounds responsible for the restoration of tissue homeostasis through the repair of damaged cells, cell proliferation, restructuring of the connective tissue and angiogenesis.

Unexplored so far is whether estrogen anti-inflammatory activity is extended to this second phase of the native inflammatory reaction. The aim of our study was to evaluate the extent to which estrogens participate in the intrinsic or T-cell-regulated resolution of the macrophage pro-inflammatory polarization stage. The study shows that estrogens interact with the signaling responsible for the onset of the resolution phase and are able to shorten the duration of the pro-inflammatory stage and to direct the resolution of the inflammation toward the acquired deactivation stage. These findings may be relevant particularly for the identification of novel estrogen receptor modulators efficacious in the prevention or mitigation of chronic inflammation.

## Results

### 7β-estradiol (E2) opposes interleukin 4 (IL4) induced alternative activation in macrophages

**Figure 1:**
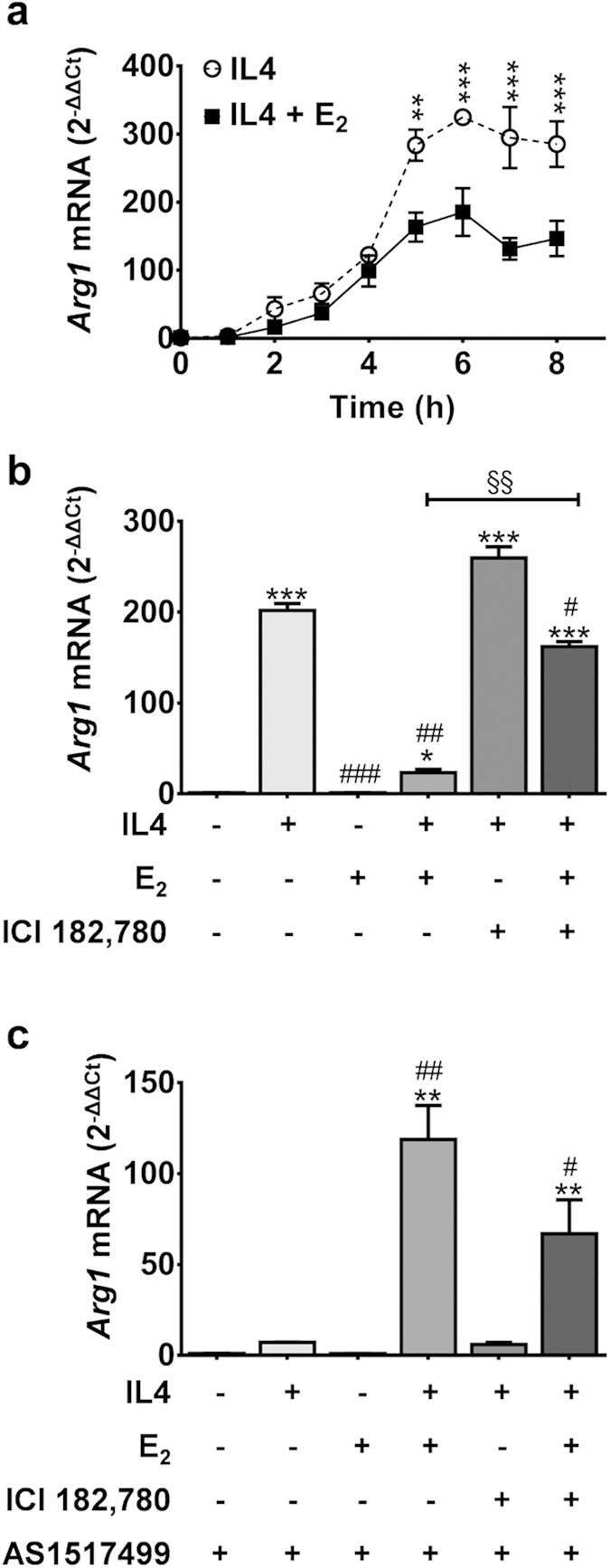
IL4 induced production of Arg1 mRNA is regulated by estrogens in Raw 264.7 cells. (**a**) Time-course of *Arg1* mRNA accumulation after IL4. RAW 264.7 were treated with 20 ng/ml recombinant mouse interleukin 4 (IL4, dashed line) alone or with 5 nM 17β-estradiol (IL4+E2, continuous line). mRNA cell content was measured by quantitative RT-PCR and data are expressed as 2^−ΔΔCt^ using the 36B4 transcript as an internal reference standard. Dots represent mean ± s.e.m. of three experiments done in triplicate, **P < 0.01; ***P < 0.001 by unpaired *t*-test. (**b**,**c**) *Arg1* mRNA was measured in cells treated for 5 hours with IL4 (20 ng/ml), E_2_ (5 nM), ICI 182,780 (250 nM), AS1517499 (1 μg/mL); data were calculated and are expressed as in the previous panel. Bars represent the mean ± s.e.m. of 6 experiments done in triplicate. *P < 0.05; **P < 0.01; ***P < 0.001 by two-way ANOVA *versus* control. ^#^*P* < 0.05; ^##^*P* < 0.01; ^###^*P* < 0.001 by two-way ANOVA *versus* IL4. §§*P* < 0.01 by two-way ANOVA.

IL4 has been described as one of the principal mediators of the transition of macrophages toward the resolution stage of inflammation. We therefore investigated the potential involvement of estrogens in the response of the mouse leukemic cell line RAW 264.7 of monocyte lineage to IL4. In our experimental paradigm, cells were stimulated at time 0 with 20 ng/ml IL4 alone or in the presence of 5 nM E_2_ and then harvested every hour in the following 8 h to measure the content of mRNA encoding arginase 1 (ARG1), a well-established marker of the AA. The results in Fig. 1a indicate that the cells responded quite rapidly to IL4 stimulation alone. Five hours after treatment with the cytokines, the content of *Arg1* mRNA was 292-fold higher than at time 0 and in the control, untreated cells (not shown). In the cells incubated with IL4 and E_2_, the response was significantly reduced, and at 5 h, the concentration of *Arg1* mRNA measured was approximately half (58%) with respect to the cells stimulated with IL4 only. Similar results were observed with another AA marker, *Chi3l3*, or *Ym1* (Supplementary Fig. S1a). In further experiments, harvesting the cells 5 h after treatment showed that E_2_ inhibition of the response to IL4 was antagonized efficiently by co-incubation with the high affinity, specific antagonist of intracellular ERs ICI 182,780 (Fulvestrant) at a 250 nM concentration. At this concentration ICI 182,780 alone did not affect *Arg1* expression (not shown). The antagonism by ICI 182,780 demonstrated the involvement of ERα or ERβ in the E_2_ interference of IL4-driven expression of the AA polarization marker (Fig. 1b). These results were confirmed using another macrophage cell line, J774A.1 (as shown in Supplementary Fig. S1b–e), suggesting that these findings were not restricted to RAW264.7. Measurement of ERα (*Esr1*) and ERβ (*Esr2*) mRNA showed that the expression of the ERα isoform is predominant in this cell line (Supplementary Fig. S2a). The expression of ERα in RAW cells was also assessed by means of immunocytochemistry and confocal microscopy, showing that ERα protein accumulates in the nuclear compartment following the treatment with 5 nM E_2_ (Supplementary Fig. S2d). All together, these results led us to conclude that the E_2_ interference of IL4 activity had to be ascribed to the liganded ERα.

### STAT 6 is not a direct target of estrogen activity

**Figure 2:**
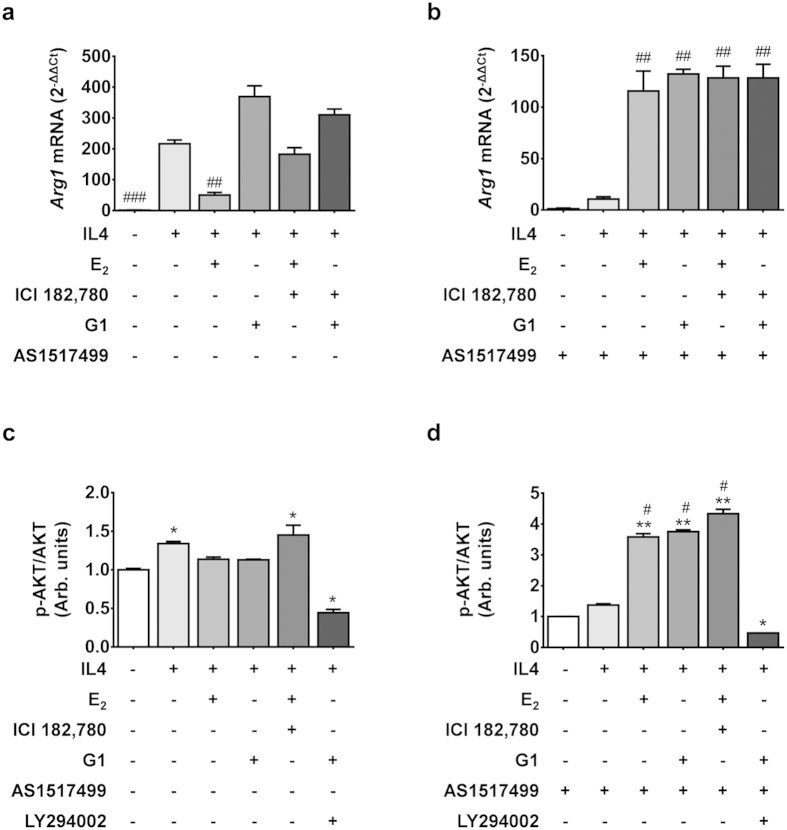
Effects of GPR30 stimulation on IL4-induced Arg1 expression. (**a**,**b**) Quantitative RT-PCR analysis of the abundance of *arginase 1* transcripts (*Arg1*) in RAW 264.7 at 5 h following treatment with IL4 (20 ng/ml), E_2_ (5 nM), ICI 182,780 (250 nM), G1 (5 nM), and AS1517499 (1 μg/mL). Results are expressed as 2^−ΔΔCt^ using the 36B4 transcript as an internal reference standard. Bars represent the mean and s.e.m. of three separate experiments carried out in triplicate. **P < 0.01 by two-way ANOVA *versus* IL4 treated cells. (**c**,**d**) The ratio of the relative quantification of phosphorylated (pAKT ) and total (AKT) intracellular Akt proteins measured by means of flow cytometry, as described in the methodology section. Cells were treated as in panels a and b. Bars represent the media of the expression measured in 10,000 cells, normalized to controls of three separate experiments carried out in triplicate. *P < 0.05; **P < 0.01 by two-way ANOVA *versus* untreated cells. ^#^*P* < 0.05 by two-way ANOVA *versus* IL4 only.

Several authors showed that the IL4 receptor (IL4R) couples with the Signal Transducer and Activator of Transcription 6 (STAT6) and activates the transcription of several genes, including the *Arg1*^18^. To verify whether the E_2_ effect on the accumulation of *Arg1* mRNA induced by IL4 was associated with an interference of the E_2_-ER complex with STAT6 signaling, we studied the effect of E_2_ in cells treated with the small molecule selective inhibitor of STAT6, AS 1517499. The blockage of STAT6 activity confirmed the major role played by STAT6 in the IL4 signal transduction because, in the presence of AS 1517499, no significant induction of the *Arg1* gene expression was observed after IL4 treatment. Nevertheless, AS 1517499 produced a paradox effect in cells treated with IL4 and E_2_ combined; in this case, E_2_ not only failed to reduce but actually significantly increased *Arg1* mRNA (119-fold induction) (Fig. 1c). The observation that this latter induction was not antagonized by ICI 182,780 led us to hypothesize the contribution of an ERα-independent pathway and to investigate the involvement of an alternative mediator of E_2_ action, the transmembrane receptor GPR30, expressed in RAW 264.7 cells at a high concentration^19^. Figure 2a shows that the specific agonist of GPR30, G1 (5 nM), did not antagonize the induction of *Arg1* mRNA by IL4 and the addition to the medium of ICI 182,780 did not have any influence on *Arg1* mRNA expression. This result was expected because the compound is not an antagonist of GPR30^20^. G1 was *per se* devoid of any effect on *Arg1* basal expression (not shown). Conversely, in the presence of AS 1517499, the exposure to IL4 + G1 led to an increase of *Arg1* mRNA superimposable with the effect of IL4 + E_2_ (Fig. 2b), and such increase was not modulated by ICI 182,780. Moreover, the treatment with the GPR30 specific agonist G15 abolished the effect of IL4 + E_2_ and IL4 + G1 on *Arg1* mRNA in the presence of AS 1517499 (Supplementary Fig. S3). Taken together, these results suggested that GPR30 was accountable for the STAT6-independent induction of *Arg1* expression previously observed following treatment with E_2_. This hypothesis was supported by the observed effects of the different treatments on the phosphorylation of AKT, a target of PI3K involved in the intracellular signaling of GPR30^21^. In the absence of STAT6 inhibitor (Fig. 2c), G1 did not further affect the increased phosphorylation of AKT induced by IL4 (+190%); however, in the presence of AS 1517499, both E_2_ and G1 had a significant effect on pAKT accumulation which, compared to IL4 alone, was highly increased (+84%). This effect was not blocked by ICI 182,780 (Fig. 2d). Blockade of AKT phosphorylation was observed in the presence of the selective inhibitor of PI3K, LY294002. No changes were observed in total AKT content. These results pointed to a complex interplay between IL4 and E_2_ in the RAW 264.7 cells, thus suggesting a significant role of the hormone in the induction of the resolution phase in this cell line.

### IL4 up-regulates the expression of ERa

Next, we investigated the extent to which the expression of ERα, ERβ and GPR30 were sensitive to IL4R activation (Supplementary Fig. S2a). QPCR showed that, at the C_t_ selected for the relative measure of the concentration of ERα and GPR30 (*Gper1*) mRNAs, no ERβ amplicon fluorescence was observed and *Gper1* mRNA content was 227% higher than ERα mRNA; however, ERα mRNA was significantly increased (+180%) after IL4 treatment and reached an intracellular concentration comparable to *Gper1*. Treatment with E_2_ and ICI 182,780 did not further change ERα mRNA content. None of the treatments had a significant effect on *Gper1* mRNA. Similarly, when we measured ERα protein content (Supplementary Fig. S2b), we observed a significant increase of ERα in the presence of IL4 (+136%). When E_2_ and/or ICI 182,780 were tested in combination with IL4, E_2_ did not influence its receptor content, which was decreased in the presence of ICI 182,780. This latter observation was in line with previous reports on the ability of ICI 182,780 to increase ERα protein turnover^22^. AS 1517499 prevention of ERα up-regulation by IL4 indicated the requirement of STAT6 to increase the ERα gene expression (Supplementary Fig. S2c).

These results provided a potential explanation for the opposite effects of E_2_ on IL4-induced *Arg1* expression: in the presence of a functional STAT6 pathway, ERα content was significantly increased by IL4, leading E_2_ to act predominantly through ERα, which limited the expression of the M2 marker. When STAT6 was inhibited, IL4 treatment was not followed by the up-regulation of ERα and E_2_ preferred signaling pathways through GPR30, which conceivably had a positive effect on *Arg1* transcription.

### Mechanism of E_2_-ERα inhibition of IL4-dependent activation of Arg1 transcription.

**Figure 3:**
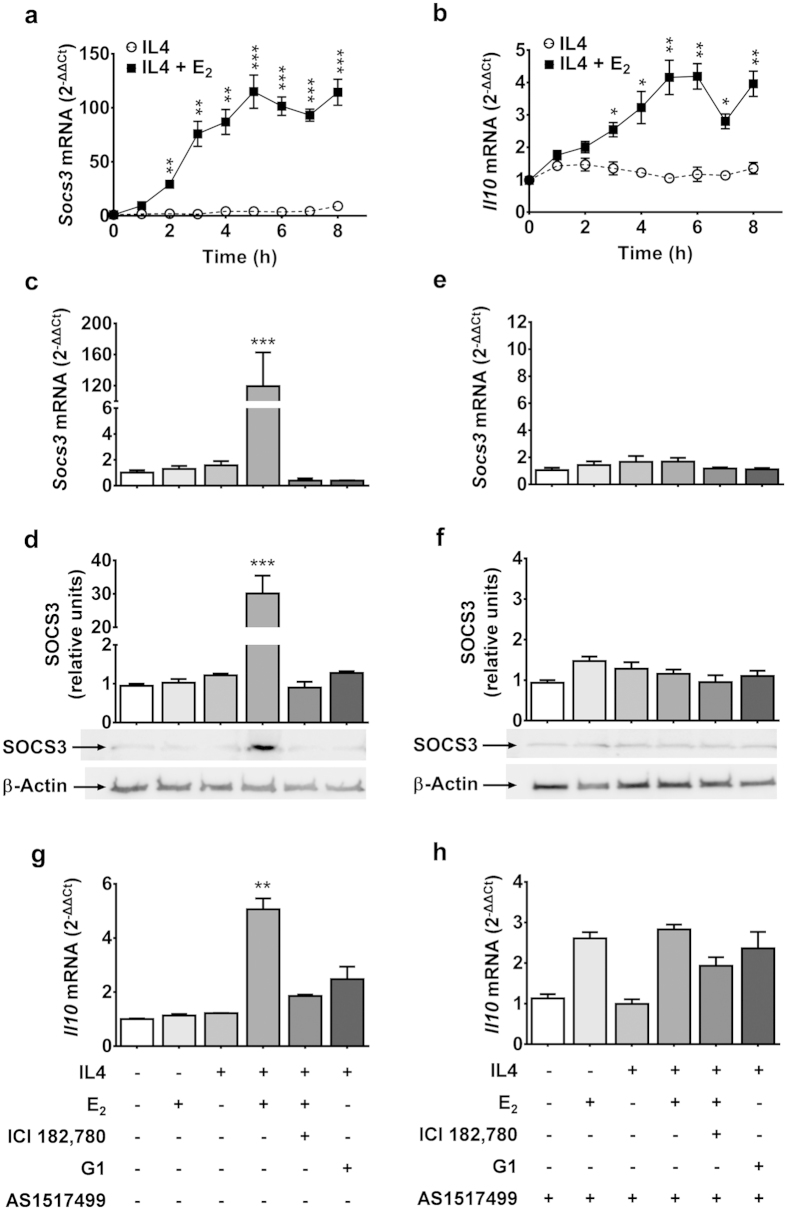
The E2-ERα complex regulates SOCS3 and Il10 synthesis in the presence of IL4 stimulation. (**a**,**b**) Time-course of *Socs3* (panel a) and *IL10* (panel b) mRNA accumulation after IL4. RAW 264.7 were treated with 20 ng/ml recombinant mouse interleukin 4 (IL4, dashed line) alone or with 5 nM 17β-estradiol (IL4+E2, continuous line). mRNA cell content was measured by quantitative RT-PCR and data are expressed as 2^−ΔΔCt^ using the 36B4 transcript as an internal reference standard. Dots represent mean ± s.e.m. of three experiments done in triplicate, *P < 0.05; **P < 0.01; ***P < 0.001 by unpaired *t*-test. (**c**–**h**) RAW 264.7 cells were treated as described in Fig. 2. (**c**,**e**) Quantitative RT-PCR analysis of mRNAs encoding for Suppressor of cytokine signaling 3 (*socs3*); data are expressed as 2^−ΔΔCt^ using the 36B4 transcript as internal reference standard. Bars represent the mean and s.e.m. of three separate experiments made in triplicate. ***P < 0.001 by two-way ANOVA *versus* untreated cells. (**d**,**f**) Representative immunoblots of RAW 264.7 lysates probed with anti-bodies against SOCS3, and subjected to densitometric analysis. β-actin served as an internal loading control. Densitometry values were normalized in controls. The bars represent the mean and s.e.m. of the densitometric analysis of three separate experiments. ***P < 0.001 by two-way ANOVA *versus* untreated cells. (**g**,**h**) Quantitative RT-PCR analysis of *Interleukin 10* mRNAs (*Il10*); data are expressed as 2^−ΔΔCt^ using the 36B4 transcript as an internal reference standard. Bars represent the mean and s.e.m. of three separate experiments made in triplicate. **P < 0.01 by two-way ANOVA *versus* untreated cells.

In several cells, including RAW 264.7, prior studies showed that the E_2_-ERα complex may directly increase the expression of the suppressor of cytokine signaling 3 (SOCS3)^23^ and an ERE motif was found at -1493–1489 nt upstream of the *Socs3* start site^24^. SOCS are a family of proteins that are highly relevant for the negative feedback that regulates cytokine signal transduction^25^. In macrophages, IL4 was described to decrease *Socs3* mRNA and protein concentrations^26^ in association with the increase of *Arg1* expression^27^, and in AA/AD-activated macrophages, the expression of SOCS1 is critical for the *Arg1*-induced anti-inflammatory response^26^. A time course study showed that IL4 did not alter *Socs3* expression profile which was significantly increased by IL4+E_2_ starting from 2 h after exposure to the hormone. The highest concentration of *Socs3* was reached at 5 h, and remained unchanged up to 8 h (Fig. 3a). Treatment with E_2_ alone at 5 h did not cause any change in SOCS3 expression. This is in contrast with prior reports^23^, likely because of the very high concentration of E_2_ used by Liang *et al.* (20-fold higher). SOCS3 expression was massively induced by IL4+E_2_ treatment, as SOCS3 mRNA and protein increased 119-fold and 30-fold, respectively (Fig. 3c,d). The effect of the sex hormone was blocked by ICI 182,780 and by the inhibition of the STAT6 pathway with the AS 1517499 compound (Fig. 3e,f). We also studied *Socs1* mRNA expression, which was increased after the treatment with IL4 only, while the combined treatments with IL4+E_2_, IL4+E_2_+ICI, and IL4+G1 blocked IL4 activity on the gene (Supplementary Fig. S4). Thus, E_2_, unable to modulate SOCS3 in unstimulated RAW 246.7, caused a major production of SOCS3 mRNA and protein in the presence of IL4. The blockage of such an effect by ICI 182,780 suggested the implication of ERα. The observation that STAT6 inhibitor prevented any E_2_-dependent increase of SOCS3 mRNA and protein provided further support for the hypothesis that, in the presence of IL4, E_2_ preferential signaling was *via* ERα.

We also verified whether other pathways could be involved in E_2_ inhibition of IL4-induced *Arg1* synthesis. Prior studies in tumor cells showed that ERα may cross-couple with the JAK/STAT3 pathways^28^. Indeed, Supplementary Figure S5 shows that, in RAW 264.7 cells, the phosphorylated form of STAT3 was slightly increased by IL4 (1.4-fold versus controls), possibly because pSTAT3 expression was already quite high prior treatments. When IL4 was combined with E_2_, the pSTAT3 was two-fold higher than in controls, and such an effect was antagonized by ICI 182,780. STAT3 is known to regulate the expression of genes involved in the resolution phase of inflammation, such as *Il10*. We therefore tested whether the effect of estrogens was associated with an induction of this cytokine. Figure 3b shows a time-dependent induction of *IL10* only in cells treated with IL4+E_2_. Figure 3g,h confirm that E_2_ was indeed able to induce *Il10* mRNA when administered in the presence of IL4. The observation that ICI 182,780 and AS 1517499 blocked the effect of the hormone suggested that its action occurred via the ERα and required fully functional IL4R signaling.

All together, these data demonstrated that E_2_, by modulating the effect of IL4 signaling, had the potential to interfere with the resolution phase driven by T lymphocytes.

Studies in wound-healing and in primary macrophage cells have clearly shown that macrophages have intrinsic mechanisms for the conversion from the pro-inflammatory to an anti-inflammatory phenotype^17^,^29^; therefore, we next investigated the capacity of E_2_ to interfere with macrophage intrinsic signaling leading to the extinction of the M1 phenotype.

### The reduced NF-κB life-span suggests a faster resolution of inflammation in the presence of E_2_ in RAW 264.7 cells

To investigate the evolution of the inflammatory process in time, we generated a reporter gene; i.e., the pNF-κB-*Luc2*-ires-*Egfp* (Supplementary Fig. S6a), which is able to gauge the state of transcriptional activity of NF-κB, the master regulator of the expression of most of the cytokines responsible for the initial phase of acute inflammation. The reporter promoter was conceived after a thorough analysis of the data in the literature and an extended experimental testing as described in the methodology section. Briefly, to identify the best NF-κB responsive element sequences, we analyzed a panel of genes involved in innate immunity (Supplementary Table S1) and thus identified two major groups of NF-κB consensus sequences. From these two groups, we selected two sequences for each group (called 1A, 1B and 2A, and 2B, Supplementary Table S2) that were particularly enriched in the promoter region of genes involved in innate immunity. Studies carried out in transient as well as in stably transfected cells using the NF-κB reporter demonstrated that activation of the reporter genes was selective, as it occurred in the presence of a number of pro-inflammatory stimuli but not the anti-inflammatory IL4 (Supplementary Figs S6b–e).

**Figure 4:**
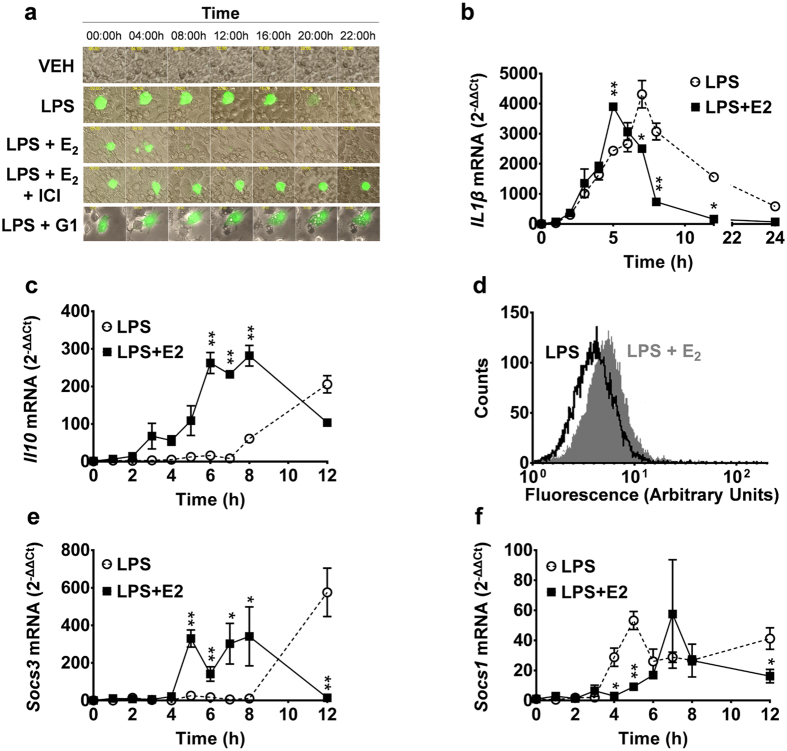
Estradiol promotes a faster M1-M2 transition in RAW 264.7 cells. (**a**) Time-lapse fluorescence microscopy of RAW264.7 transiently transfected with the NFkB-*Luc2*-ires-*Egfp* reporter vector, stimulated for 4 h with LPS (1 μg/ml), and then treated with E_2_ (5 nM), ICI 182,780 (250 nM), G1 (5 nM), and IL4 (20 ng/ml). The panel represents time-lapse movies. Frames were captured every 30 min. NF-κB activity is reported by the expression of EGFP (green). Original magnification, ×40. Data representative of six independent experiments. (**b**,**c**) mRNA expression profile of *Interleukin 1*β mRNA (*Il1*β) and *Interleukin 10* (*Il10)* measured by quantitative RT-PCR in RAW264.7 treated with 1 μg/ml LPS (LPS) alone or in the presence of 5 nM 17β-estradiol (LPS+E2). (**d**) Flow-cytometry histograms of fluorescence associated to LPS-FITC engulfed in RAW 264.7 at 6 hours after stimulation with LPS-FITC (LPS, 1 μg/ml) and co-treated with E_2_ (5 nM). Fluorescence intensity, detected using the FL1 (515 ∼ 545 *nm*) photodetector, is represented in arbitrary units, logarithmic scale. Black line: LPS; gray area: LPS+E_2_. Data representative of triplicate experiments. (**e**,**f**) mRNA expression profile of *Suppressor of cytokine signaling 3* (*Socs3) and Suppressor of cytokine signaling 1* (*Socs1)* assessed with real-time RT-PCR analysis in RAW 264.7 treated as in panel b. (**b**,**c**,**e**,**f**) Data are expressed as 2^−ΔΔCt^ using the *36B4* transcript as an internal reference standard and represent the mean and s.e.m. of triplicate experiments. *P < 0.05; **P < 0.01 by unpaired *t*-test. Statistical significance *versus* untreated is reported in Supplementary Information.

Using this reporter system, we studied the influence of E_2_ on the course of the inflammatory process in RAW 264.7 cells transfected with the NF-κB reporter and treated with lipopolysaccharide (LPS, 1 μg/mL) to trigger the inflammatory response. The production of the EGFP fluorescence was observed as early as 4 h following LPS treatment. From this time point on, the intracellular fluorescence was monitored continuously by means of time-lapse microscopy (Fig. 4a). The cells expressed the fluorescent protein for 22 ± 2 h after LPS; at this time point, the fluorescence disappeared rapidly. At approximately 2 h before the disappearance of the fluorescence, we observed membrane blebbing and release of fluorescent vesicles in the medium (Supplementary Fig. S7). When the RAW 264.7 cells were treated with LPS for 4 h and then with 5 nM E_2_, the duration of fluorescence expression was significantly shortened: at 8 ± 2 h, the cells ceased to express fluorescence after having undergone morphological changes similar to those observed in the cells not exposed to E_2_ (Supplementary Fig. S7). The accelerated loss of EGFP was not observed in cells exposed to both E_2_ and ICI 182,780 or to the GPR30 agonist G1 alone.

These data indicated that the hormone receptor complex was able to reduce the time of NF-κB transcriptional activity induced in response to a strong inflammatory stimulus, suggesting the ability of the hormone to shorten the pro-inflammatory phase, possibly by anticipating the resolution of inflammation. However, these findings were not in line with the prior described effect of the E_2_-ERα complex, which appeared to limit the ability of IL4 to induce markers of AA polarization. This led us to verify whether the termination of EGFP fluorescence in cells treated with E_2_ was associated with the functional changes characteristic of an accelerated loss of the M1 phenotype and conversion to AA/AD. To this aim, we investigated the time course of expression of two cytokine hallmarks for either the early phase of the pro-inflammatory process (IL1β) or the initiation of the resolution phase (IL10)^30^. Figure 4b shows that, in RAW 264.7 cells, 2 h after LPS treatment, the content of *Il1*β mRNA started to accumulate to reach its maximal concentration at 7–8 h (4300-fold *versus* T = 0); from this time on, the content of cytokine mRNA slowly decreased and at 24 h after LPS was still significantly higher than in untreated cells (585-fold). When cells were co-treated with LPS and E_2_, the changes in time of *Il1*β mRNA concentrations were quite different, as the max concentration was reached at 5 h after LPS (3,900-fold *versus* T = 0) and the decrease was much steeper than in the LPS-treated cells. By the 12 h, *Il1*β mRNA content was not significantly different than untreated cells. When we focused on the trigger of the resolution phase of the inflammatory response, i.e., IL10, we observed that, in cells treated with LPS alone, the *Il10* mRNA started to accumulate at approximately 8 h after the inflammatory insult (60-fold *versus* T = 0) and was 205-fold higher at 12 h than at time 0. When cells were exposed to E_2_ and LPS together, the profile of *Il10* mRNA changed significantly as the cells started to accumulate *Il10* much earlier than in the previous treatment (60-fold *versus* T = 0 at 3 h), and the highest amount of *Il10* mRNA (+253-fold *versus* T = 0) was measured at 6–8 h after treatment (Fig. 4c).

Further support for our findings was provided by the observed effects exerted by E_2_ on phagocytosis, another marker of the pro-inflammatory phenotype and a crucial event for the establishment of the resolution phase of the inflammatory process^31^. Figure 4d shows the flow cytometry analysis of RAW 264.7 cells treated for 6 h with 1 μg/ml FITC-conjugated LPS in the presence or absence of E_2_. The engulfment of the fluorescent compound at 6 h was significantly higher (+38%) in cells treated with LPS+E_2_ than in controls receiving only the pro-inflammatory stimulus. These data supported the hypothesis of a faster onset and evolution of the M1 inflammatory phase in the presence of the sex hormone.

We finally tested how, in this treatment paradigm, the presence of E_2_ was influencing *Socs* gene expression. As expected, in cells treated with LPS alone, the accumulation of *Socs3* mRNA started late in the process and was clearly measurable only at 12 h after LPS (590-fold *versus* T = 0, Fig. 4e). The presence of E_2_ in the medium significantly accelerated the time in which this mRNA started to accumulate (360-fold *versus* T = 0 at 5 h after LPS+E_2_). This latter effect was restricted in time, as the cells had ceased to accumulate *Socs3* mRNA 12 h after the treatment (Fig. 4e). The maximum expression of *Socs3* mRNA at 6 h was consistent with prior observations on genes directly regulated by the E_2_-ERα complex^32^,^33^. *Socs1* mRNA was present in the cells at a concentration much lower than *Socs3* mRNA, and its production could not be clearly associated to a specific phenotype (Fig. 4f). As expected, no IL4 mRNA was synthesized by the macrophagic cell line at any time.

## Discussion

The present study provided novel insights on the anti-inflammatory effects of estrogens, potentially relevant to the understanding of the role of estrogen deprivation in the evolution of pathologies characterized by a high degree of inflammation. This study, aimed at evaluating the effect of estrogens on the resolution of inflammation, indeed showed that the activation of the intracellular estrogen receptor shortened the pro-inflammatory phase and influenced the extrinsic and intrinsic programs leading to the resolution of the inflammatory phase, favoring the progression of macrophages toward the IL10-dependent AD phase, which is responsible for immunomodulation and tissue remodeling.

**Figure 5:**
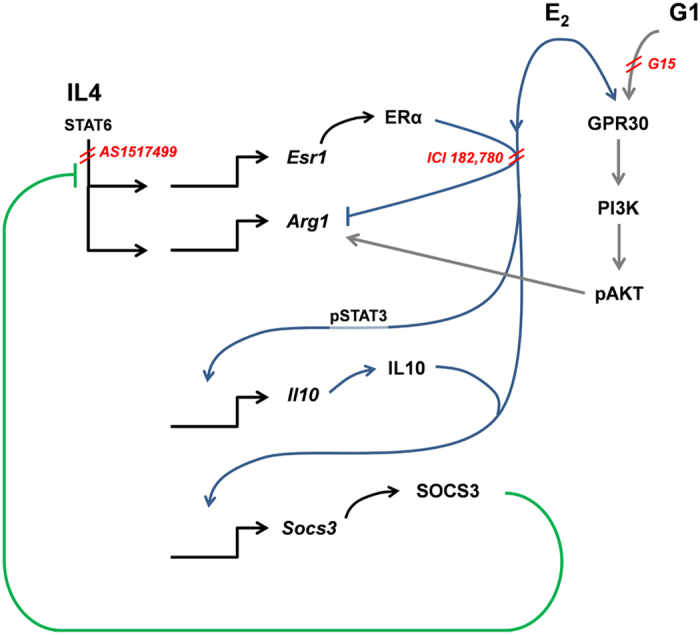
Representative scheme of the proposed mechanism.

The mechanisms involved in estrogen-dependent progression of the inflammatory response are multiple: in the absence of stimulation by other immune cells, estrogens preferentially bind the GPR30 receptor and activate the synthesis of *Arg1* mRNA through the PI3K-AKT pathway. However, when IL4R is stimulated, the intracellular concentration of ERα is increased, and in the presence of E_2_, this receptor activates *Socs3* expression, leading to the blockage of IL4R signaling^27^. At the same time, the E_2_-ERα complex facilitates STAT3 phosphorylation and the activation of the *Il10* promoter, as also described in cells of different lineage^34^,^35^. Interestingly, after the inflammatory insult, E_2_ appeared to significantly accelerate the onset of the AD polarization by hastening IL1β synthesis and anticipating the production of IL10 and SOCS3 even in the absence of IL4. Conceivably, in this case, the ERα activity might be facilitated by intrinsic factors involved in the response to the inflammatory stimulus (Fig. 5).

The finding that IL4 treatment induces an increase in the expression of ERα (Supplementary Fig. S2) is in agreement with previous reports^36^,^37^. In particular, the observation of a transient up-regulation of ERα mRNA in human macrophages following the treatment with IL4/IL13^37^ suggested that this IL4 effect is well conserved in mammals. This phenomenon appeared to be functionally relevant, as the increased content of ERα is associated with a decrease in the production of ARG1, the main function of which is related to the inhibition of the pro-inflammatory response in the inflammatory process. The evidence of a negative effect of E_2_ on *Arg1* expression (Fig. 1a,b) in cells stimulated by IL4 is therefore quite surprising, being in apparent contrast with estrogen anti-inflammatory activity. Yet, in the inflammation process, the resolution phase is multifaceted^38^; in addition to the up-regulation of the genes, which is a hallmark of the AA phenotype, such as *Arg1*, and necessary for the diminution of the activity of the inflammatory molecules produced, other cytokines (e.g., IL10) are generated to repair the damaged tissues in the AD phenotype. Indeed, IL10 was proposed as the marker of the AD phenotype^39^ characterized by pro-healing functions with the release of angiogenetic factors, such as vascular endothelial growth factor-A (VEGFA) and transforming growth factor beta (TGFβ), which are essential for tissue repair^40^.

Our present results indicate that E_2_, by activating the intracellular ERα, shortens the time spent by macrophages in the inflammatory status and facilitates the onset of the AD phenotype. Our findings are in line with and provide mechanistic insights to the reports of an estrogen positive effect on angiogenesis and proliferation^41^,^42^,^43^ or in wound healing of chronic wounds^44^,^45^.

Our study may impact the understanding of the causes for the increased incidence of chronic inflammatory disorders in women after the cessation of estrogen production by the ovaries. Growing experimental evidence points to a relevant role of macrophage polarization in the progression of several pathologies linked to climaterium, the M1 pro-inflammatory state associated with the progression of osteoporosis, atherosclerosis and metabolic diseases, including diabetes^46^,^47^,^48^. Therefore, the ability of E_2_ to promote the resolution phase of inflammation may be important for the protective effects exerted by the hormone prior to menopause.

The effects of estrogens on macrophage polarization may, however, also have a negative impact on health; in fact, in young women, the incidence of pathologies driven by the AA/AD macrophages, such as scleroderma^49^ and asthma^50^, is significantly higher than in post-menopause^51^,^52^. Moreover, in selective types of cancer, tumor-associated macrophages (TAM) produce IL10 and TGFβ, contributing to the general suppression of anti-tumor activities^53^. It is well known that E_2_ stimulates proliferation and metastatic potential of ERα positive breast cancer cells^54^, and because tumor progression and resistance to chemotherapy are associated with elevated levels of SOCS3^55^ and STAT3^56^, it is tempting to speculate that the effects of the estrogen on tumor macrophages may be involved in E_2_-dependent cancer growth.

The observation that estrogen accelerates the inflammatory process, thus promoting the natural resolution of inflammation, suggests an important aspect to be fully exploited for new hormone replacement therapies. Indeed, the current trend in the generation of anti-inflammatory therapies is to create drugs able to facilitate the resolution of inflammation^57^ in order to avoid the collateral effects of classical anti-inflammatory compounds, which may expose the organism to opportunistic infections^58^. These novel compounds would diminish the extent of chronic inflammation, thus limiting the associated tissue damage. Estrogen, via the mechanism here described, would represent an ideal anti-inflammatory treatment for women in post-menopause.

## Methods

### Reagents

Unless otherwise specified, chemicals were purchased from Merck (Darmstadt, Germany), and culture media and additives were obtained from Life Technologies-Invitrogen (Paisley, Scotland, United Kingdom). E_2_, LPS (isotype 0.111:B4), LPS-FITC (isotype 0.111:B4), LY294002, and ICI 182,780 were obtained from Sigma (Milan, Italy). Recombinant mouse IL4 was obtained from PeproTech (London, UK). AS 1517499 was obtained from Axon Medchem BV (Groningen, The Netherlands). G1 and G15 were obtained by Tocris (Bristol, UK).

### Cell cultures

RAW 264.7 and J774A.1 cells were purchased from the American Type Culture Collection (Manassas, Va.) and grown in red-phenol free Dulbecco minimal essential medium (DMEM)-10% DCC serum (DCC; Celbio, EuroClone, Milan, Italy) supplemented with 2 g of sodium carbonate/liter, 0.11 g of sodium pyruvate/liter, and streptomycin-penicillin (50,000 IU plus 50 mg per liter) in a humidified 5% CO2–95% air atmosphere at 37 °C.

### Real Time Reverse transcription (RT)-PCR

RNA was extracted from RAW 264.7 cells. All samples were resuspended in TRIzol reagent (Invitrogen, Milan, Italy), and RNA was isolated according to the manufacturer’s instructions. One microgram of RNA was used for cDNA preparation with Moloney murine leukemia virus reverse transcriptase (Promega, Milan, Italy), as previously described^7^. Control reactions without the addition of the enzyme were performed for each sample. A 1:4 cDNA dilution was amplified using SYBR green chemistry. The PCR was carried out in triplicate or duplicate on a 96-well plate using GoTaq®qPCR Master Mix technology (Promega) according to the manufacturer’s protocol using a 7900HT fast real time PCR system (Applied Biosystems, Life Technologies) with the following thermal profile: 2 min at 95 °C; 40 cycles, 15 s at 95 °C, 1 min at 60 °C. Gene expression of target genes was assessed for *Arginase-1* (*Arg1*; forward primer, 5′-CAGAAGAATGGAAGAGTCAG-3′; reverse primer, 5′-CAGATATGCAGGGAGTCACC-3′), *Esr1* (forward primer, 5′-GAAGAGTTTGTGTGCCTCAAAT-3′; reverse primer, 5′-GTGCCGGATATGGGAAAGGATG-3′), *Esr2* (forward primer, 5′-CCACACCATCCTCAATCACTAC-3′; reverse primer, 5′-CAGTAACAAGGGCATGGAAC-3′), *Gper1* (forward primer, 5′-CGGCACAGATCAGGACACCC-3′; reverse primer, 5′-TGGGTGCATGGCAGAAATGA -3′), *Il1*β (forward primer, 5′-TGCCACCTTTTGACAGTGATG-3′; reverse primer, 5′-GCTGCGAGATTTGAAGCTGG-3′), *Il10* (forward primer, 5'-GGTTGCCAAGCCTTATCGGA-3′; reverse primer, 5′- ACCTGCTCCACTGCCTTGCT-3′), *Socs3* (forward primer, 5′- GGAGATTTCGCTTCGGGACT-3′; reverse primer, 5′- CAGATATGCAGGGAGTCACC -3′), *Socs1* (forward primer, 5′- CTCGCTCCTTGGGGTCTGTTG -3′; reverse primer, 5′- GCGTGCTACCATCCTACTCGA -3′), *Il4* (forward primer, 5′- CGGCATTTTGAACGAGGTCA -3′; reverse primer, 5′- CTCTGTGGTGTTCTTCGTTGC -3′).

Data were analyzed using the 2^−ΔΔCt^ method^59^ using *36b4* as the housekeeping gene (forward primer, 5′-GGCGACCTGGAAGTCCAACT-3′; reverse primer, 5′-CCATCAGCACCACGGCCTTC-3′).

### Western Blotting

Cells were lysed in a buffer for total cellular extracts, containing 5 mM MgCl_2_, 20 mM HEPES (pH 7.9), 420 mM NaCl, 0.1 mM EDTA, 20% glycerol, 0.1% Triton, 5 mM β-mercaptoethanol, 0.1 mM PMSF, 10 μg/ml aprotinin, and 1 μg/ml leupeptin. The lysates were frozen on dry ice for 5 min and then thawed at 37 °C for 5 min for three times. The samples were centrifuged at 13,000 rpm at 4 °C for 20 min and the supernatants were collected and stored at −20 °C. Protein concentration was estimated by a Bradford protein assay using BSA as the standard. Equal amounts of protein (20 μg) were dissolved in Laemmli’s sample buffer, boiled for 5 min, and separated with a SDS-polyacrylamide minigel (10% and 7.5% polyacrylamide for Ym1 and CD206 detection, respectively) and then transferred overnight at 15 mA into 0.45 μm Hybond-ECL membrane (GE Healthcare, Milan, Italy). Membranes were incubated for 1 h with blocking solution containing 5% (w/v) nonfat milk in Tris-buffered saline (TBS) and subsequently probed for 1 h at room temperature with rabbit antibodies anti-mouse STAT3 (1:500; #12640,Cell Signaling Technologies, USA), pSTAT3 Tyr705 (1:500; #9138, Cell Signaling Technologies, USA), STAT6 (1:1000; #9362, Cell Signaling Technologies, USA), pSTAT6 Tyr641 (1:500; #9361, Cell Signaling Technologies, USA), ERα (1:500; SC-542, Santa Cruz Biotechnologies, USA), and SOCS3 (1:1000; #2923, Cell Signaling Technologies, USA) in incubation solution (TBS containing 5% (w/v) nonfat milk and 0.1% Tween 20. After extensive washing in TBST (TBS + 0.1% Tween 20), blots were incubated with horseradish peroxidase-conjugated goat anti-rabbit IgG (1:2000; PI-1000, Vector Labs, USA) in incubation solution, for 1 h at room temperature. After extensive washing in TBST, immunoreactive bands were visualized using a chemiluminescence assay detection system according to the manufacturer’s instructions (Amersham™ ECL™ Western Blotting Analysis System, GE Healthcare). To ascertain that blots were loaded with equal amounts of protein lysates, they were also incubated in the presence of the antibody against β-actin protein (1:10,000; Sigma-Aldrich Corp., Milan, Italy). Subsequently, for semiquantitative analyses, the densities of the protein bands of STAT3 and pSTAT3 (86 kDa), STAT6 and pSTAT6 (110 kDa), ERα (66 kDa), SOCS3 (26 kDa), and β-actin (42 kDa) were measured by densitometric scanning of the membrane with Gel Doc™ XR Imaging Densitometer (Bio-Rad Lab, Segrate, Italy) with a specific software program (Image Studio 4.0, LI-COR Inc.).

### PI3-Kinase/Akt activity assay

Modulation of the PI3-Kinase/*Akt* pathway has been assessed using FlowCellect™ PI3K Activation Dual Detection Kit (FCCS025105, Merk-Millipore, USA) a two color flow cytometry kit designed to measure the extent of Akt phosphorylation relative to the total Akt expression in a given cell population, according to the manufacturer’s protocol. The kit includes two directly conjugated antibodies, a phospho-specific Anti-phospho-Akt (Ser473)-Alexa Fluor 488 and an Anti-Akt-Alexa Fluor 647 conjugated antibody to measure total levels of Akt. The levels of both the total and phosphorylated protein can be measured simultaneously in the same cell, resulting in a normalized and accurate measurement of Akt activation after stimulation. Briefly, at the end of the different treatments, cells were put on ice, washed three times with PBS, detached by scraping, suspended in 500 μl PBS. The cells in suspension were pipetted several times to achieve a single cell suspension. Equal volume of ice-cold Fixation Buffer has been added to the cell suspension, followed by a 20-minute incubation on ice. Cells were spun at 670 × g for 5 minutes in a 4 °C centrifuge, supernatant was aspirated, and the pellet was washed twice with the Wash Buffer. Cells were permeabilized by adding 100 μL of ice-cold Permeabilization Buffer and incubated on ice for 20 minutes. Cells have been washed twice with the Wash Buffer and resuspended in 90 μL of assay buffer. For staining, 5 μL of Anti-phospho-Akt-Alexa Fluor 488 and 5 μL of Anti-Akt-Alexa Fluor 647 were added to each sample. Cells were incubated for one hour on ice in the dark, washed twice and resuspended in 200 μl Assay Buffer. The median fluorescence of 10,000 cells for each sample was analyzed with a flow cytometry system (BD FACSCalibur, Becton Dickinson Biosciences, San Jose, CA, USA).

### Assessment of LPS internalization

RAW 264.7 macrophage cells (approximately 2 × 10^5^) were stimulated with 1 μg/ml of FITC-labeled LPS for 1 h and then treated with vehicle or 17β-estradiol (5 nM). Internalization of FITC-LPS was monitored at 3 h and 6 h by measuring the mean fluorescence of 10,000 cells for each sample with a flow cytometry system (BD FACSCalibur, Becton Dickinson Biosciences, San Jose, CA, USA); cells stimulated with unlabeled LPS served as autofluorescence reference.

### Construction of the pNFκB-*Luc2*-ires-*EGFP* plasmid

Each functional cassette of the vector NFκB-*Luc2*-ires-*EGFP* was flanked with unique restriction sites to facilitate further manipulations. Each element was sequentially cloned, using standard cloning procedures, in the p*EGFP*-N1 (Clontech Laboratories, Inc., 1290 Terra Bella Ave. Mountain View, CA 94043 USA): 1) 641-bp DNA fragment encoding IRES element excised from pIRES vector (Clontech Laboratories, Inc., 1290 Terra Bella Ave. Mountain View, CA 94043 USA) and cloned into the SacII/AgeI restriction sites; 2) 1642-bp DNA fragment encoding *luc2* excised from the pGL4.10 basic vector (Promega Corp., Madison, WI) and cloned into the the SalI/SacII restriction sites; 3) 220-bp DNA fragment of NF-κB responsive elements and TATA minimal promoter (Supplementary Fig. S6) excised from the NF-κB1 vector (see below) and cloned into the AseI/SaII. The construct NF-κB1 was obtained by cloning into the AseI/SalI restriction sites of pBluescript II KS+(Strategene, Agilent Tecnologies, Santa Clara, United States) four selected NF-κB responsive elements, synthetized by Eurofins Genomics, Ebersberg Germany. Then, the 220-bp DNA fragment of NF-κB responsive elements and TATA minimal promoter was excised and cloned into the AseI/SaII restriction sites in the pGl2 basic vector (Promega Corp., Madison, WI) in order to obtain NF-κB1. The constructs NF-κB1.2, NF-κB1.3, NF-κB1.4, NF-κB1.5, NF-κB1.6 and NF-κB1.7 were derived from NF-κB1 by sequential partial digestion in order to obtain the corresponding NF-κB responsive elements arrangements.

### Transient Transfection

RAW 264.7 cells were seeded into 24-well plates (approximately 2 × 10^5^ cells per well) and cultured overnight. Transfections were carried out using the X-tremeGENE HP DNA transfection reagent (Roche), following the manufacturer’s instructions. Two μg of pNF-κB-*Luc2*-ires-*Egfp* were added into 200 μl red-phenol free DMEM (Celbio, EuroClone, Milan, Italy) before mixing with 6 μl of X-tremeGENE HP DNA transfection reagent. The plasmid DNA/X-tremeGENE HP mixture was incubated at room temperature for 25 min and then added into each well of the 24-well plates. Cells were incubated for 48 hours in a humidified 5% CO_2_-95% air atmosphere at 37 °C before treatments.

### Time Lapse fluorescence microscopy

Culture medium was removed and substituted with 1 ml of fresh medium. Cells were treated with LPS (1 μg/ml), incubated for 4 h in a humidified 5% CO_2_-95% air atmosphere at 37 °C, and then co-treated with vehicle, 17β-estradiol (5 nM), G1 (5 nM), ICI 182,780 (250 nM), or IL4 (20 ng/mL). Time-lapse images were recorded at 40x magnification in phase contrast and in fluorescence using a GFP filter (Excitation wavelength, 457–487 nm; emission wavelength, 502–538 nm) using a Zeiss Axiovert 200 M microscope (Carl Zeiss), beginning right after the co-treatment and ending after 24 h. 24-well plates were inserted in a cell cultivation system consisting of a heated microscope stage, incubator box, atmosphere controlling device (CTI controller 3700, Carl Zeiss) equipped with a humidifier, and a temperature controlling device (Tempcontrol 37–2 digital, Carl Zeiss). The CTI controller 3700 supplied the cultivation chamber with a heated mixture of CO_2_/air. The CO_2_ concentration and the temperature were continuously monitored and regulated by a digital feedback control algorithm, enabling a regulated dispersion of CO_2_ into the air stream and steady heating. Images were acquired every 30 min at each of the 24 positions with an AxioCam digital camera (Carl Zeiss) controlled by the AxioVision 4.2 software (Carl Zeiss).

### Luciferase enzymatic assay

Supernatants containing luciferase were collected and total protein concentration was determined with a Bradford assay. Luciferase enzymatic activity in tissue extracts was then assayed with a luciferase assay buffer (470 μm luciferine, 20 mm Tricine, 0.1 mm EDTA, 1.07 mm (MgCO_3_)_4_·Mg(OH)_2_ × 5H_2_O; 2.67 mm MgSO_4_ × 7H_2_O in H_2_O, pH 7.8, with 33.3 mm DTT and 530 μm ATP). The light intensity was measured with a luminometer.

### Statistical analysis

Unless otherwise stated, statistical significance was assessed by an unpaired t-test or two-way ANOVA using Bonferroni’s multiple comparison post hoc tests that were performed with GraphPad Prism 5 (GraphPad Software).

## Additional Information

**How to cite this article**: Villa, A. *et al.* Estrogen accelerates the resolution of inflammation in macrophagic cells. *Sci. Rep.* **5**, 15224; doi: 10.1038/srep15224 (2015).

## Supplementary Material

Supplementary Information
