# Supplementary material for: Estrogen accelerates the resolution of inflammation in macrophagic cells
Source: Sci Rep. 2015 Oct 19;5:15224. doi: 10.1038/srep15224 (PMC4609992; doi:10.1038/srep15224)
Supplement: Supplementary Information [file srep15224-s1.doc]

**Title: Estrogen accelerates the resolution of inflammation in macrophagic cells**

**Authors: Alessandro Villa^1^, Nicoletta Rizzi^1^, Elisabetta Vegeto^1^, Paolo Ciana^1^, Adriana Maggi^1^**

**Affiliation:** ^1^Center of Excellence on Neurodegenerative Diseases and Department of Pharmacological and Biomolecular Sciences, University of Milan, Milan, Italy.

**Supplementary information.**

**Supplementary materials and methods.**

**ERα immunofluorescence and confocal laser scan microscopy.**

RAW264.7 cells were grown in 24-well plate on glass coverslips for 2 days, then fixed for 10 min in 4% paraformaldehyde in 0.1 M PBS (pH 7.5) at room temperature. Cells were washed three times with PBS and incubated for 30 min at room temperature with Blocking solution (10% goat serum, 1% BSA, 0.5% Tween-20 in PBS). Cells were incubated with 400 μl/glass coverslip in a PBS solution containing 1:500 dilution of the anti-human ERα monoclonal antibody (1D5, from Zymed Lab, San Francisco, CA, USA) and 1% goat serum, o/n at 4 °C. Cells were washed three times in PBS and incubated with the secondary antibody Alexa-fluor 488 goat-anti mouse form Molecular Probes (Leiden, The Netherlands) for 60 min at RT. After 3× 5 min washes in PBS, coverslips were mounted with a mixture 50% PBS + 50% glycerol. ERα immunofluorescence was imaged with a Radiance 2100 confocal laser scanning microscope (Biorad, Milan, Italy) based on a Eclipse TE2000-S Microscope (Nikon, Milan, Italy) and operating in the simultaneous acquisition mode. Images were taken at the magnification of 180×.

**Supplementary figure legends.**

**Figure 4. Estradiol promotes a faster M1-M2 transition in RAW 264.7 cells** (b) Statistical significance *versus* untreated : LPS, 2 h *P* < 0.05; 3 h-5 h *P* < 0.01; 6 h *P* < 0.05; 7 h-8 h *P* < 0.01; 12 h *P* < 0.001; 24 h *P* < 0.001. LPS + E_2_, 2 h-3 h *P* < 0.05; 4 h *P* < 0.01; 5 h *P* < 0.001; 6 h *P* < 0.05; 7 h-8 h *P* < 0.01. (c) Statistical significance *versus* untreated: LPS, 8 h *P* < 0.05; 12 h *P* < 0.01. LPS + E_2_, 4 h-5 h *P* < 0.05; 6 h-8 h *P* < 0.01; 12 h *P* < 0.01. (e) Statistical significance *versus* untreated: LPS, 12 h *P* < 0.05. LPS + E_2_, 5 h-6 h *P* < 0.01; 7 h-8 h *P* < 0.05; 12 h *P* < 0.01. (f) Statistical significance *versus* untreated: LPS, 4 h *P* < 0.05; 5 h *P* < 0.01; 6 h-8 h *P* < 0.05; 12 h *P* < 0.05. LPS + E_2_, 5 h-6 h *P* < 0.05; 12 h *P* < 0.01; 8 h *P* < 0.05; 12 h *P* < 0.05.

**Figure S1. IL4-induced synthesis of *Arg1* and *Chi3l3* mRNA is regulated by estrogens in Raw 264.7 and J774A.1 cells.** (a) Quantitative RT-PCR analysis of the abundance of *Chi3l3* transcripts in RAW 264.7 at 5 h after treatment with IL4 (20 ng/ml) and E_2_ (5 nM). Data are expressed as 2^-ΔΔCt^ using the 36B4 transcript as an internal reference standard. Bars represent the mean and s.e.m. of three separate experiments made in triplicate. *, *P* < 0.05 by two-way ANOVA *versus* untreated cells. (b,c) Quantitative RT-PCR analysis of the abundance of *Arg1* transcripts in J774A.1 cells treated for 5 hours with IL4 (20 ng/ml), E2 (5 nM), ICI 182,780 (250 nM), AS1517499 (1 μg/mL); data were calculated and are expressed as in the previous panel. Bars represent the mean ± s.e.m. of 2 experiments done in triplicate. *, *P* < 0.05; ***, *P* < 0.001 by two-way ANOVA versus control. #, P < 0.05; ###, P < 0.001 by two-way ANOVA versus *IL4*. §§, P < 0.01 by two-way ANOVA. (d,e) Quantitative RT-PCR analysis of the abundance of *Chi3l3* transcripts in J774A.1 cells treated for 5 hours with IL4 (20 ng/ml), E2 (5 nM), ICI 182,780 (250 nM), AS1517499 (1 μg/mL); data were calculated and are expressed as in the previous panel. Bars represent the mean ± s.e.m. of 2 experiments done in triplicate. **, *P* < 0.01; ***, *P* < 0.001 by two-way ANOVA versus control. ##, P < 0.01; ###, P < 0.001 by two-way ANOVA versus *IL4*. §§, P < 0.01 by two-way ANOVA.

**Figure S2.** **ERα mRNA and protein are significantly increased after IL4 treatment in** **RAW 264.7.** Cells were treated for 5 h with IL4 (20 ng/ml), E_2_ (5 nM), ICI 182,780 (250 nM) and AS1517499 (1 μg/mL) and then harvested and the lysates used for quantitative RT-PCR or Western blot as described in the methodology section. (a) Quantitative RT-PCR comparative analysis of *Esr1* (ERα), *Esr2* (ERβ) and *Gper1* (GPR30) mRNA accumulation. The slopes of the standard curves carried out to determine the efficiency of the primers done with serial dilutions of the cDNA were -3.4225 (R^2^=0.9982) for *Gper1* and -3.4932 (R^2^=0.9964) for *Esr1*; the calculated efficiency (Efficiency = 10^-1/slope^) was 1.96 (97.9%) for *Gper1* and 1.93 (96.7%) for *Esr1* thus allowing the direct comparison of Ct (Schmittgen TD, Nat Protoc 2008;3:1101-8). Data are expressed as 2^-ΔΔCt^ using the *36b4* transcript as internal reference standard. Bars represent the mean and s.e.m. of three separate experiments carried out in triplicate. *, *P* < 0.05; **, *P* < 0.01 by two-way ANOVA *versus* *Esr1* mRNA in untreated cells. (b,c) Immunoblots were probed with anti-bodies against ERα and densitometric analysis was carried out. β-actin served as internal loading control. Densitometry values were normalized in controls. Bars represent the mean and s.e.m. of three separate experiments. *, *P* < 0.05 by two-way ANOVA *versus* untreated cells. (d) Confocal image of ERα protein detected in RAW cells by immunocytochemistry performed in the presence of vehicle (left panel) or 10^−9^ M 17β-estradiol (right panel) added for 30 min, by using ERα specific primary antibody.

**Figure S3. Effects of GPR30 inhibition on IL4-induced Arg1 expression.** (a,b) Quantitative RT-PCR analysis of the abundance of *arginase 1* transcripts (*Arg1*) in RAW 264.7 at 5 h following treatment with IL4 (20 ng/ml), E_2_ (5 nM), G1 (5 nM), G15 (250 nM), and AS1517499 (1 μg/mL). Results are expressed as 2^-ΔΔCt^ using the 36B4 transcript as an internal reference standard. Bars represent the mean and s.e.m. of three separate experiments carried out in triplicate. ***, *P* < 0.001 by two-way ANOVA *versus* IL4 treated cells.

**Figure S4. IL4-induced upregulation of Socs1 is blocked by E_2_, ICI, and G1.** Quantitative RT-PCR analysis of the abundance of *Suppressor of cytokine signaling 1* (*Socs1)* transcripts in RAW 264.7 at 5 h following treatment with IL4 (20 ng/ml), E_2_ (5 nM), ICI 182,780 (250 nM), and G1 (5 nM). Data are expressed as 2^-ΔΔCt^ using the 36B4 transcript as an internal reference standard. Bars represent the mean and s.e.m. of three separate experiments made in triplicate. ***, *P* < 0.001 by two-way ANOVA *versus* untreated cells.

**Figure S5. IL4 and E_2_ induce STAT3 phosphorylation.** Upper panel: representative immunoblot of RAW 264.7 lysates of RAW 264.7 cells at 5 h following treatment with IL4 (20 ng/ml), E_2_ (5 nM), and ICI 182,780 (250 nM), probed with anti-bodies against STAT3 and phosphorylate STAT3. β-actin served as internal loading control. Middle and lower panels: the bars represent the mean and s.e.m. of the densitometric analysis of three separate experiments. Densitometry values were normalized in controls. *, *P* < 0.05 by unpaired *t*-test *versus* control.

**Figure S6.** **Design and validation of the NFkB-*Luc2*-ires-*Egfp* construct.** (a) Schematic representation of NFkB-Luc2-ires-EGFP construct with the nucleotide sequences of 2A, 2B, 1A and 1B responsive elements. (b) Relative luciferase activity represented as relative light units/μg of protein, measured by means of luminometry in a NFkB-Luc stably transfected RAW264.7 cell line. Cells were treated with the following conditions: 20% FBS for 24 hours; 0.4 M sorbitol for 24 hours; 810 ng/ml of TNFα for 6 hours, 270 ng/ml of Il1β for 6 hours, 2.5 µg/µl of LPS for 6 hours, 1 µM dexamethasone for 6 hours, 1 µM dexamethasone pre-treatment for 1 hours and 2.5 µg/µl of LPS for 6 hours. At the end of the treatments, the cells were collected and luciferase activity was measured by enzymatic assay. Each bar represents the mean and s.e.m. of relative light unit on total µg of proteins measured in triplicate. *, P < 0.05; **, P < 0.01; ***, P < 0.001 by by two-way ANOVA *versus* vehicle. (c) Relative luciferase activity represented as relative light units/μg of protein, measured by means of luminometry in NFkB-Luc stably transfected RAW264.7 cell line. Cells were treated with 2.5 µg/µl LPS and with increasing doses of IL4. Each bar represents the mean and s.e.m. of relative light units on total µg of proteins measured in triplicate. (d) Evaluation of responsiveness of NFkB-*Luc2*-ires-*Egfp* construct in three cell lines. Luciferase enzymatic activity was measured on total protein extracts, respectively, from RAW264.7, NIH-3T3 and BV-2 cell lines transiently transfected with NFkB-*Luc2*-ires-*Egfp* reporter vector and treated with LPS (2.5 μl/µl) or IL4 (20 ng/ml). Each bar represents the mean and s.e.m. of luciferase activity *versus* not treated sample (vehicle) measured in triplicate. (E) Evaluation of *Arginase 1* transcripts (*arg1*) by RT-PCR on RNA extracted from stable reporter cell line RAW264.7 treated as reported in supplementary Figure 4c.

**Figure S7. Disappearance of EGFP signal follows the observation of membrane blebbing.** Fluorescence microscopy of RAW264.7 cells transiently transfected with NFkB-*Luc2*-ires-*Egfp* reporter vector, stimulated for 4 h with LPS (1 μg/ml), and then treated with vehicle or E_2_ (5 nM). EGFP fluorescence (green) correlated with NF-κB promoter activity. Membrane blebbing and vesicles, observed at 15 h following the treatment with vehicle (left) or at 4 h following the treatment with E_2_ (right) are indicated by black arrows. Original magnification, ×40. Data are representative of at least six independent experiments.


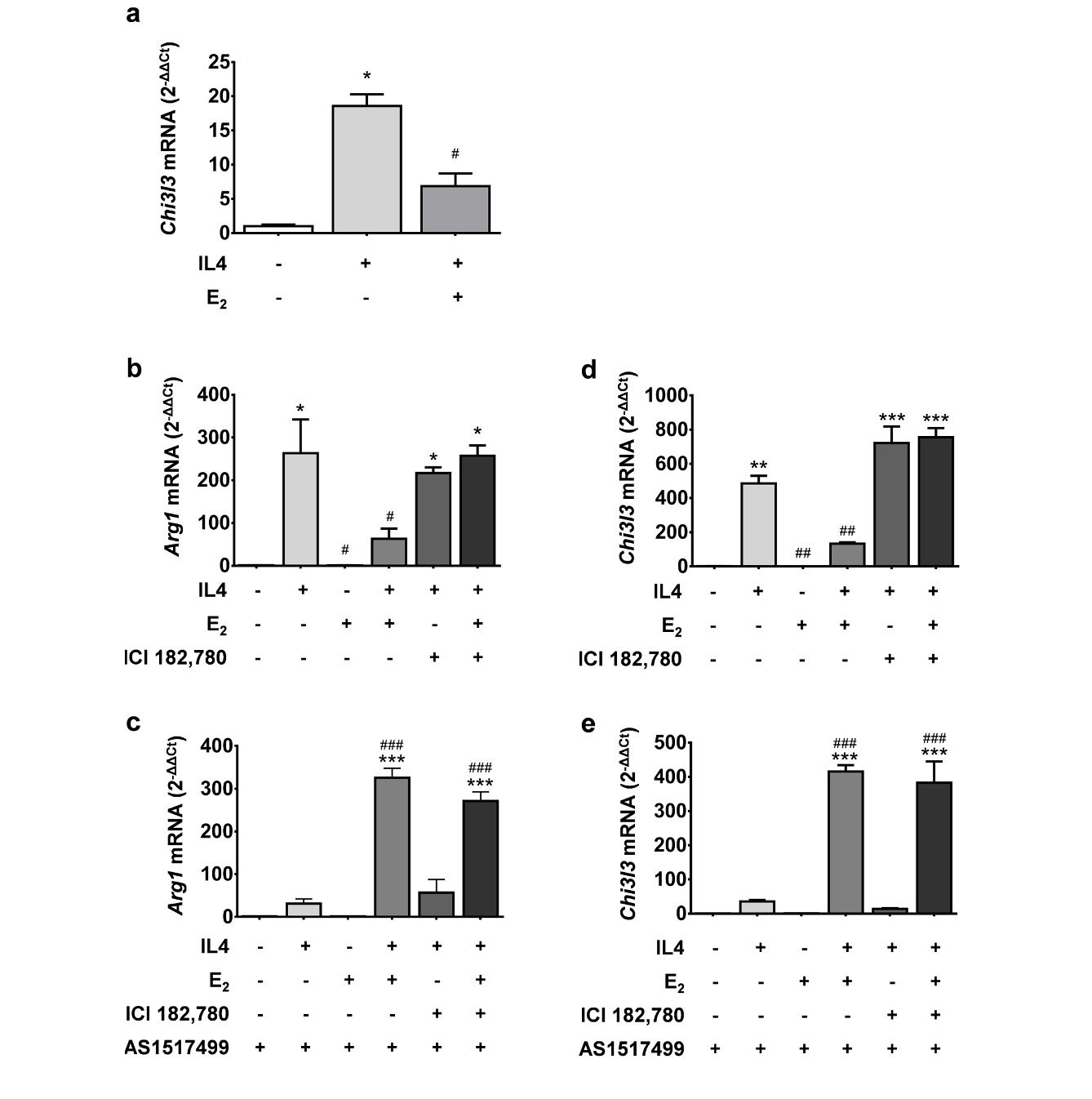


**Figure S1.**


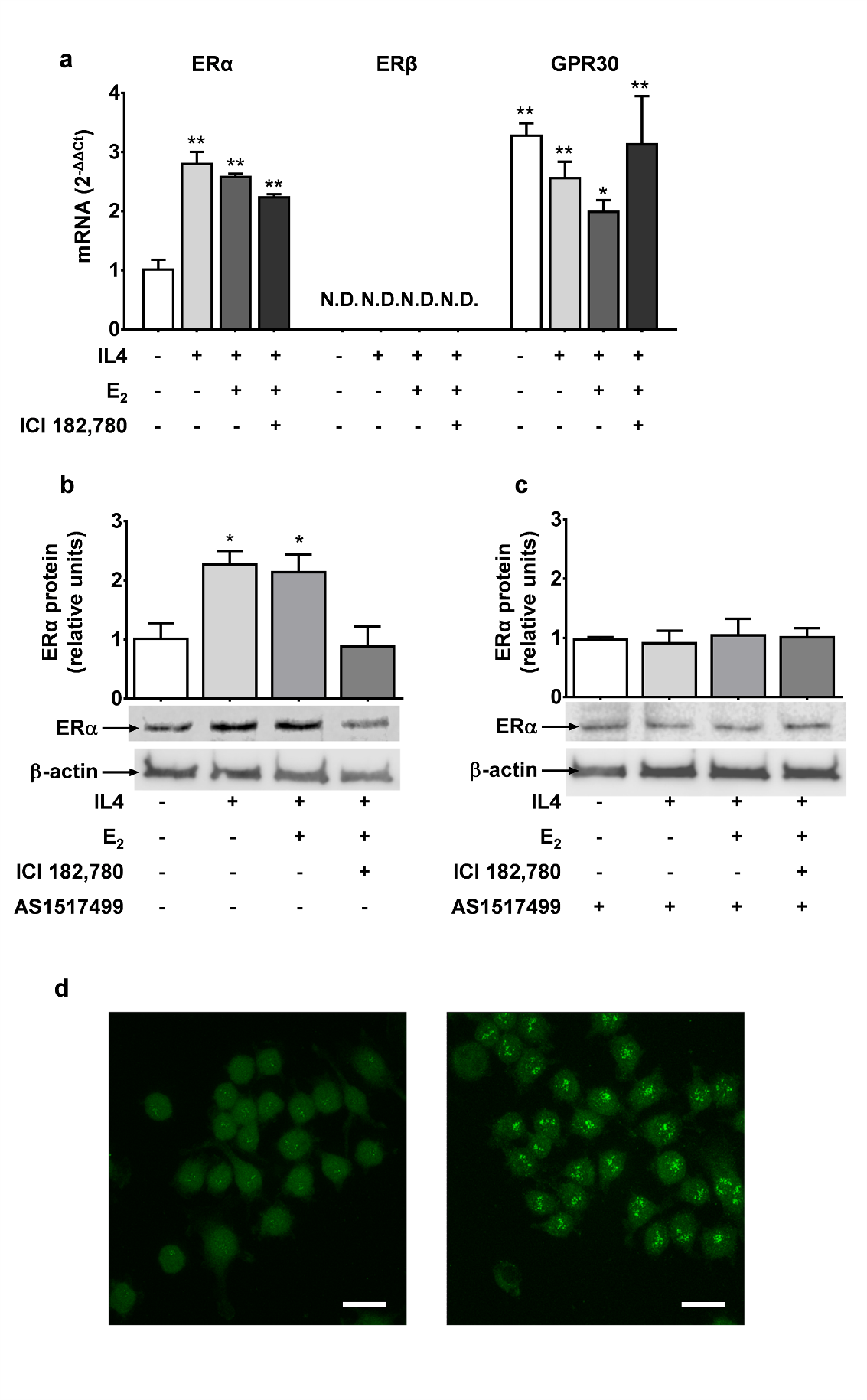


**Figure S2.**

**
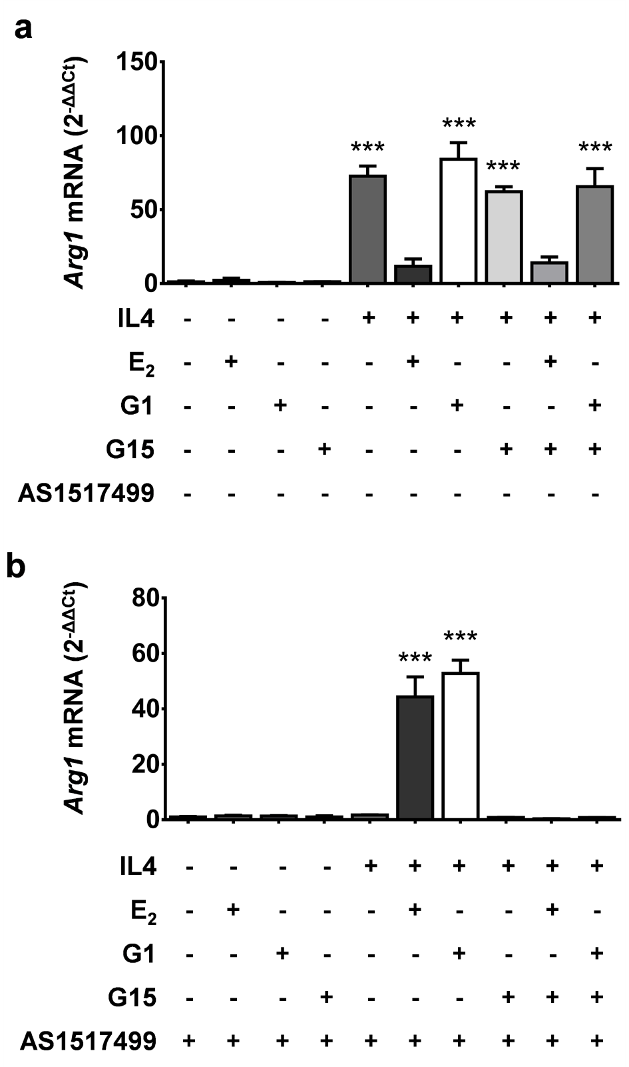
**

**Figure S3.**


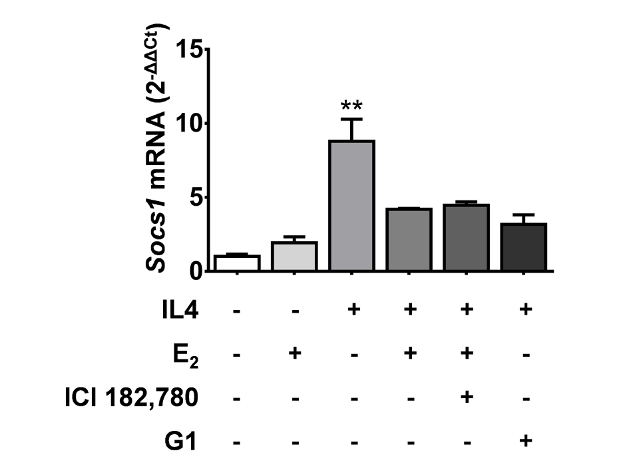


**Figure S4.**


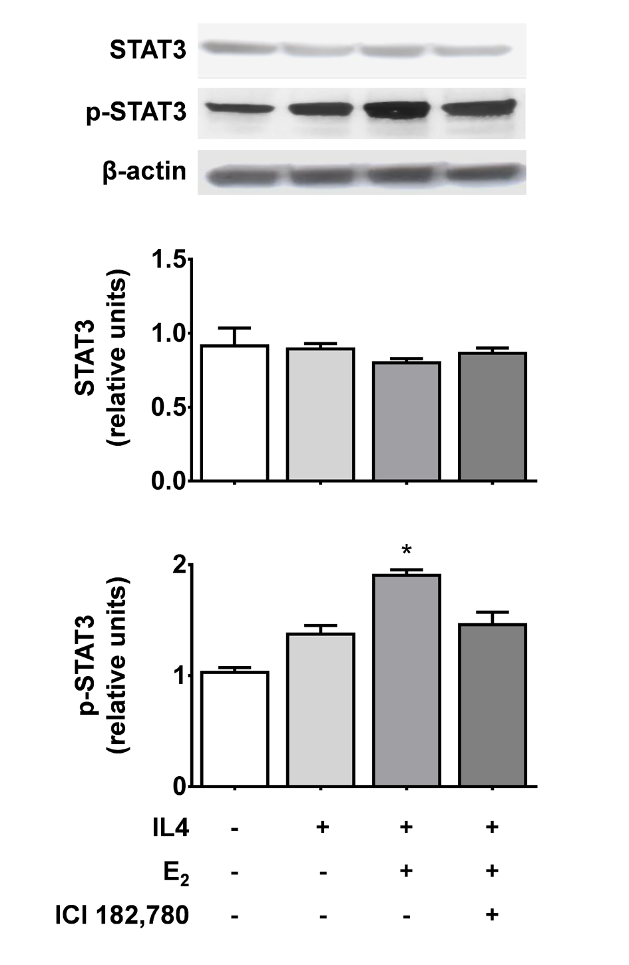


**Figure S5.**


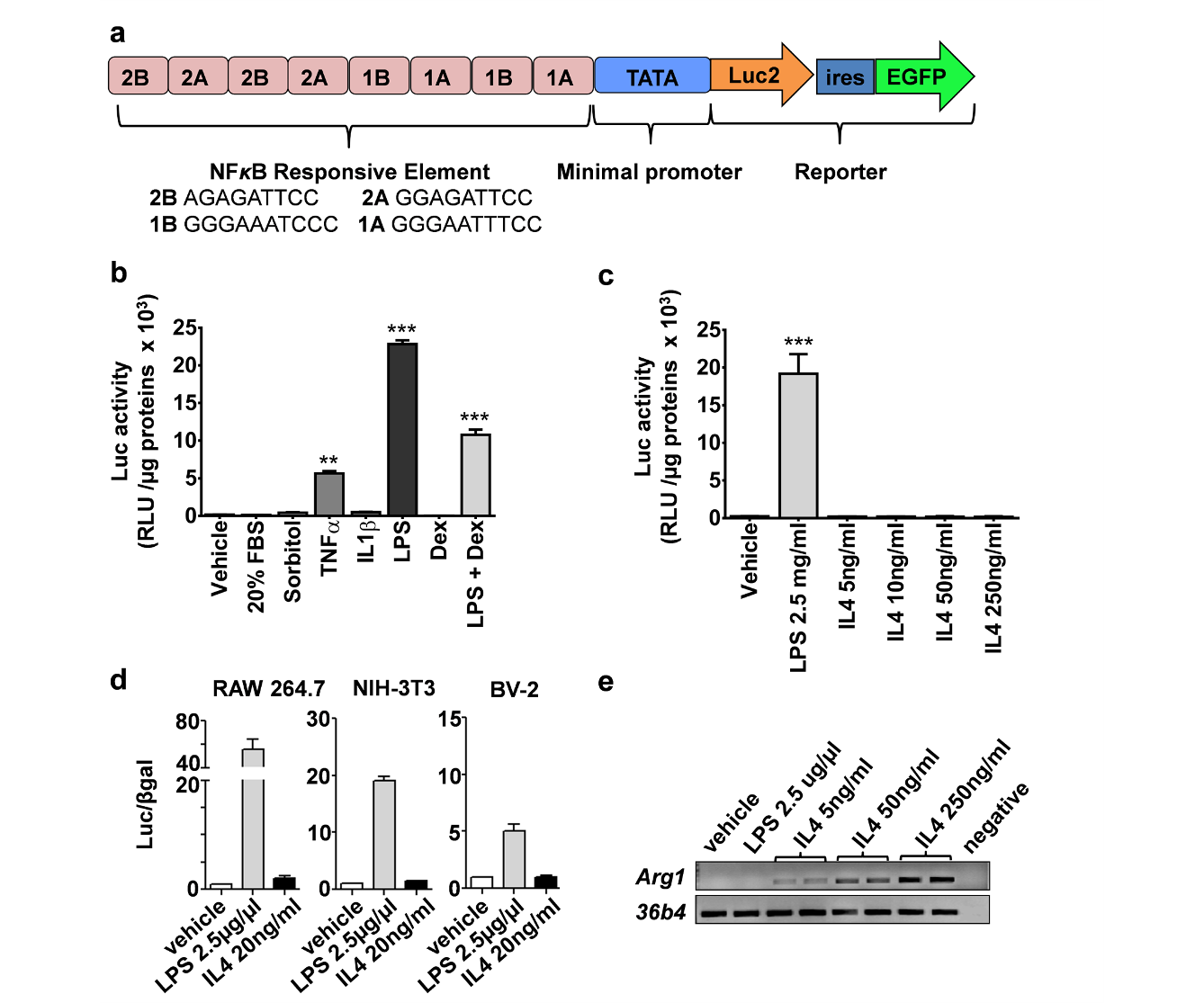


**Figure S6.**


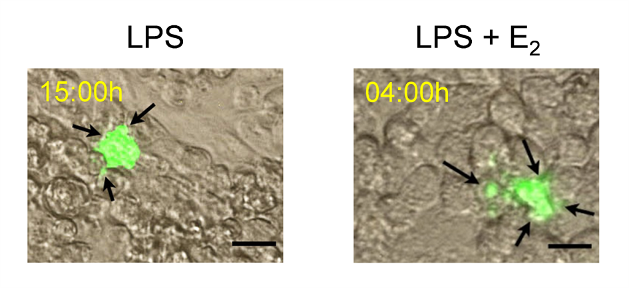


**Figure S7.**

***Table S1.*** *NF-kB responsive element in the promoter region of genes of the innate immunity*

| **Gene** | **bp from ATG** | **Consensus** | **Classifications** |
| --- | --- | --- | --- |
| CINC-1 | -62 | 1A GGGAATTTCC | Cytokines/chemokine |
| CXCL 11 | -66 | 1A GGGAATTTCC | Cytokines/chemokine |
| Gro alfa; beta; gamma (CXCL1) | -66 | 1A GGGAATTTCC | Cytokines/chemokine |
| KC | -59 | 1A GGGAATTTCC | Cytokines/chemokine |
| MIP 2 | -57 | 1A GGGAATTTCC | Cytokines/chemokine |
| IL-8 | -72 | 1A GGGAATTTCC | Cytokines/chemokine |
| CCL19 | -361 | 1A GGGAATTTCC | Cytokines/chemokine |
| MCP-1/JE | -2603 | 1A GGGAATTTCC | Cytokines/chemokine |
| ICOS | -2127 | 1A GGGAATTTCC | Cytokines/chemokine |
| TNFRSF9 | -89 -77 | 1A GGGAATTTCC | immunoreceptor |
| PLAU | -1865 | 1A GGGAATTTCC | protease |
| CCL2 | -2600 | 1A GGGAATTTCC | Cytokines/chemokine |
| IER3 | -92 | 1A GGGAATTTCC | Regulators of apoptosis |
| CARD15 | -25 | 1A GGGAATTTCC | immunoreceptor |
| KC | -60 | 1A GGGAATTTCC | Cytokines/chemokine |
| LIX | -84 | 1A GGGAATTTCC | Cytokines/chemokine |
| CXCL5 | -90 | 1A GGGAATTTCC | Cytokines/chemokine |
| CD137 | -915 | 1A GGGAATTTCC | Immunoreceptor |
| CD40 | -566 | 1A GGGAATTTCC | Immunoreceptor |
| IFN-b | -56 | 1B GGGAAATCCC | Cytokines/chemokine |
| IL-1 receptor antagonist | -84 | 1B GGGAAATCCC | Cytokines/chemokine |
| CCL15/leukotactin | -191 | 1B GGGAAATCCC | Cytokines/chemokine |
| IL-23 | -105 | 1B GGGAAATCCC | Cytokines/chemokine |
| IL-1b | -288 | 1B GGGAAATCCC | Cytokines/chemokine |
| ELF3 | -88 | 1B GGGAAATCCC | Transcription factor |
| TNFAIP3 | -239 | 1B GGGAAATCCC | Zing finger protein |
| NRGA2 | -585 | 1B GGGAAATCCC | Transcription factor |
| GM-CSF | -87 | 2A GGAGATTCC | Growth factors, ligands |
| G-CSF | -179 | 2B AGAGATTCC | Growth factors, ligands |

**Table S2.** Frequency of NFkB consensus sequences (called 1A 2A e 1B 2B) in promoter genes involved in innate immunity.

| **1A** | **G** | **G** | **G** | **A (75%)** | **A (75%)** | **T (44%)** | **T (60%)** | **T (52%)** | **C** | **C** |
| --- | --- | --- | --- | --- | --- | --- | --- | --- | --- | --- |
| **GGGAATTTCC** |  |  |  | G (25%) | G (12%) | A (32%) | G (16%) | C (42%) |  |  |
|  |  |  |  |  | T (9%) | G (16%) | A (16%) | A (8%) |  |  |
|  |  |  |  |  | C (4%) | C (8%) | C (8%) |  |  |  |
| **1B** | **G** | **G** | **G** | **A (75%)** | **A (75%)** | **T (44%)** | **T (60%)** | **T (52%)** | **C** | **C** |
| **GGGAAATCCC** |  |  |  | G (25%) | G (12%) | A (32%) | G (16%) | C (42%) |  |  |
|  |  |  |  |  | T (9%) | G (16%) | A (16%) | A (8%) |  |  |
|  |  |  |  |  | C (4%) | C (8%) | C (8%) |  |  |  |
| **2A** | **G (55%)** | **G** | **A** | **G (60%)** | **A** | **T** | **T** | **C** | **C** |  |
| **GGAGATTCC** | A (45%) |  |  | A (40%) |  |  |  |  |  |  |
| **2B** | **A (45%)** | **G** | **A** | **G (60%)** | **A** | **T** | **T** | **C** | **C** |  |
| **AGAGATTCC** | G (55%) |  |  | A (40%) |  |  |  |  |  |  |
